# Authors’ Response

**DOI:** 10.4269/ajtmh.19-0741b

**Published:** 2020-01

**Authors:** Moncef Belhassen-García, Montserrat Alonso-Sardón, Antonio Muro, Juan Luis Muñoz Bellido, Javier Pardo-Lledias

**Affiliations:** Servicio de Medicina Interna; Sección de Enfermedades Infecciosas; CAUSA; IBSAL; CIETUS; Universidad de Salamanca; Salamanca, Spain; E-mail: mbelhassen@hotmail.com; Área de Medicina Preventiva y Salud Pública; IBSAL; CIETUS; Universidad de Salamanca; Salamanca, Spain; E-mail: sardonm@usal.es; Laboratorio de Inmunología Parasitaria y Molecular; IBSAL; CIETUS; Facultad de Farmacia; Universidad de Salamanca; Salmanca, Spain; E-mail: ama@usal.es; Servicio de Microbiologia; CAUSA; IBSAL; CIETUS; Universidad de Salamanca; Salamanca, Spain; E-mail: jlmubel@usal.es; Servicio de Medicina Interna; CIETUS; Hospital Marques de Valdecilla; IDIVAL; Santander, Spain; E-mail: javipard2@hotmail.com

Dear Sir,

We have read the comments by Manciulli et al.^1^ concerning our study published recently in the journal. We appreciate their thorough revision, although we do not agree with many of their comments.

We agree that the definition of “complications of echinococcosis cyst” may be “fuzzy” for some readers. Cystic echinococcosis (CE) is usually asymptomatic, but when complications arise, their clinical presentations can be very diverse. Therefore, it is impossible to include a glossary of terms in the methodology section that contains all the clinical characteristics that these patients could have. For example, a bone fracture in a patient admitted to an emergency department can have very different causes; however, in the absence of other causes, tomography detection of CE at the fracture site suggests that the fracture is a complication of CE.

In our opinion, the suggestions by Manciulli et al. for a classification based on symptoms, including asymptomatic uncomplicated, symptomatic complicated, etc., would not be helpful because none of the CE complications in our cohort were asymptomatic. We do not consider the term “Watch and Wait” a misnomer. Manciulli et al. are probably confusing a meaning of this term frequently used in clinical practice (deciding to not treat a patient at the moment, and instead monitor the evolution of the patient’s illness) and that used in our study,^2^ with specific clinical practice guidelines. We do not understand why Manciulli and others explain the high rate of CE complications by the high rate of “Watch and Wait.” In our study, all the complicated cysts were primary, as indicated in the title of this article.

Regarding methodology, our study was retrospective and longitudinal, as described in the Methods section, and we analyzed the clinical characteristics of the patients at the time of diagnosis. Thus, patients with and without CE complications were compared to establish the risk factors for these complications. Because our report was retrospective and the criteria of selection might have involved selection bias, we recognized these limitations in our article. However, the authors of the letter criticize these limitations, yet acknowledge the difficulty of performing other types of studies in CE.^3^

We await the first clinical results from the European Registry of Echinococcosis (HERACLES), a collaborative project started in 2013 that is led by some of the authors of this letter. In the meantime, we suggest that retrospective studies may give us useful insights for managing these patients.

Another issue that Manciulli et al. had with our study was the exclusion criteria of patients with complications related to treatment. These data were not included because we had covered this topic previously.^4^

## References

[1] Manciulli T Giordani MT Lissandrin R Brunetti E Tamarozzi F, 2020 Comment on: “complications associated with initial clinical presentation of cystic echinococcosis: a 20-year cohort analysis”. Am J Trop Med Hyg 102: 241–242. 31971161 10.4269/ajtmh.19-0741a PMC6947792

[2] Teggi A, 2004 An up-to-date on clinical management of human cystic echinococcosis. Parassitologia 46: 405–407. 16044701

[3] Lissandrin R Tamarozzi F Mariconti M Manciulli T Brunetti E Vola A, 2019 Watch and wait approach for inactive echinococcal cyst of the liver: an update. Am J Trop Med Hyg 101: 628–635. 29869600 10.4269/ajtmh.18-0164 PMC6090333

[4] Velasco-Tirado V 2018 Management of cystic echinococcosis in the last two decades: what have we learned? Trans R Soc Trop Med Hyg 112: 207–215. 29897552 10.1093/trstmh/try050

